# Intraspecific differences in leaf decomposition and associated traits in closely related *Carex* species: a microcosm experiment

**DOI:** 10.1007/s00442-025-05740-1

**Published:** 2025-06-12

**Authors:** Szilvia Márta Neumann, Jules Segrestin, Marie Konečná, Aleš Lisner, Markéta Applová, Petr Blažek, Anna E-Vojtkó, Eva Janíková, Lars Götzenberger, Jan Lepš

**Affiliations:** 1 Department of Botany, Faculty of Science, University of South Bohemia, České Budějovice, Czech Republic; 2 Institute of Botany, Czech Academy of Sciences, Třeboň, Czech Republic; 3 Biology Center of the Czech Academy of Sciences, Institute of Entomology, České Budějovice, Czech Republic

**Keywords:** Litter quality, Leaf traits, Intraspecific variability, Afterlife, Environmental preferences

## Abstract

Litter decomposition is a fundamental process in carbon and nutrient cycling in terrestrial ecosystems. In a microcosm experiment we investigated the determinants of leaf decomposition with consideration of the 'afterlife’ effect hypothesis, which suggests a connection between green leaf traits and the decomposability of leaf material. We collected senesced litter and living leaves of individuals of four *Carex* species widely distributed in the Czech Republic. We aimed to determine the extent of intra- and interspecific variability in decomposition rates (*k* values), whether species ranking was consistent along environmental gradients and whether intraspecific trait variability affected litter decomposability, as we would expect from the 'afterlife’ effect hypothesis. While litter quality and decomposition rates significantly differed between fresh leaves and litter, species identity explained a prominent amount of variability in both. The effect of populations was around a tenth of species identity’s, nonetheless still significant. Environmental variables and leaf traits generally showed rather weak or non-significant correlations with decomposition rates, which suggests that within closely related species ecological preferences might not be correlated with leaf decomposability, nor the conditions of individual localities are modifying tissue quality in a way to affect decomposability. While the correlation between fresh leaf and litter decomposition rates was not very strong (r = 0.51), fresh leaves provided a fair prediction of litter decomposition. However, considering the pattern of intra- and interspecific differences in decomposition rates, and the quality of fresh leaves and litter, using litter to determine leaf decomposability might give more realistic results.

**Supplementary Information:**

The online version contains supplementary material available at 10.1007/s00442-025-05740-1.

## Introduction

Litter decomposition is a critical component of nutrient cycling and ecosystem functioning (Swift et al. 1979; Coûteaux et al. 1995) which can partly determine the structure of plant communities through its effects on nutrient availability for plants (Hättenschwiler et al. 2005). At the global scale, litter decomposition rates have been shown to be primarily determined by climatic and other abiotic conditions: under warm and humid conditions plant litter tends to decay at a faster rate than in cold and dry environments (Szefer et al. 2017). Regional conditions can also determine the composition of fungal and bacterial decomposer communities (Aerts 1997), further influencing the rate of litter decomposition. Finally, litter quality has been shown to strongly influence the rate of decomposition, especially at the local scale (Aerts et al. 2003; Cornwell et al. 2008; Bontti et al. 2009; Makkonen et al. 2012; van den Brink et al. 2023).

Litter quality can be determined by chemical and morphological traits affecting the performance of decomposers. Typically, the higher the concentrations of nutrients (e.g. nitrogen, potassium, magnesium, and calcium) and the lower the fiber concentration (i.e. cellulose, hemicellulose, and lignin), the faster the decomposition under the same environmental conditions (Bumb et al. 2018; Joly et al. 2023). As most of the aboveground litter results from the process of leaf senescence, its quality is expected to depend both on the traits of living leaves and on the resorption capacity of plants during leaf senescence (Prieto and Querejeta 2020). The relationship between foliar traits of living plants and their associated litter decomposability has been extensively studied in the last decades (e.g. Cornelissen and Thompson 1997; Fortunel et al. 2009; Kazakou et al. 2009; Freschet et al. 2010; Rosenfield et al. 2020; Rawat et al. 2020), suggesting a strong correlation between leaf traits and decomposability. Thus, leaf characteristics at the peak of growing season should reflect the decomposability of the corresponding litter. For example, the C:N ratio in fresh leaves has been frequently used as a predictor of litter decomposability (Taylor et al. 1989; Aerts 1997). In living leaves of graminoids (including *Carex*) the amount of foliar potassium—as one of the nutrients leaching immediately because of its high mobility—has been shown to promote litter decomposability (Cornelissen and Thompson 1997; Hawkesford et al. 2012). Similarly, morpho-anatomical traits of living leaves can be good predictors of decomposition; for example leaves with low leaf dry matter content (LDMC) are commonly found to have higher decomposition rates (Kazakou et al. 2006; Bumb et al. 2016).

Disentangling the relationship between green leaf characteristics and litter decomposability led to the concept of the 'afterlife’ effect hypothesis (Cornelissen et al. 2004; Mori et al. 2020), which predicts that trait responses to environmental conditions can affect the rate of litter decomposition. Fast growing species usually have higher nutrient and low fiber content in aboveground tissues. This makes them palatable and digestible, and also produces fast decomposing litter (Pálková and Lepš 2008; Bumb et al. 2016). While the relationship between response traits and decomposition has been often studied (Cornelissen 1996; Cornelissen et al. 2004; Guo et al. 2024), most studies focused on interspecific differences. In contrast, within species responses (intraspecific trait variability) remain poorly investigated (Albert et al. 2011, but see e.g. Crutsinger et al. 2009), despite the fact that species responses are known to be highly context dependent. Hence, it is unclear whether species ranking based on their trait values would be consistent under different environmental conditions (Mudrák et al. 2019), and if the expected responses in decomposition rates on the interspecific level would also apply within species. More importantly, we still lack knowledge on whether the 'afterlife’ effect hypothesis applies at the intraspecific level and if intraspecific trait variability among plant populations could affect litter quality and decomposability (Aerts 1996; Lecerf and Chauvet 2008).

To investigate the consequences of intra- and interspecific trait variability associated with different environmental conditions on litter decomposition, we sampled four species of *Carex* in different locations of the South Bohemian region of the Czech Republic. Leaf traits were measured at the peak of vegetation season in May 2020 on each population and litter was collected on the same population in November the same year to estimate their decomposability. As we were primarily interested in the consequences of litter quality on the decomposition rate, we ran a decomposition assay under controlled conditions (Taylor and Parkinson 1988). We aimed to answer the questions i) whether intra- or interspecific variability was better at explaining litter decomposability, ii) whether variation in decomposability between *Carex* populations followed the same trend along environmental gradients in the four species, and iii) whether intraspecific trait variability in response traits affected litter decomposition following the 'afterlife’ effect hypothesis. From a methodological point of view, we also asked if the decomposability of dried fresh leaves is a good indicator of litter decomposability, as collecting leaf litter can be laborious, particularly in species rich communities (Pálková and Lepš 2008). Therefore, using dried green material for decomposition experiments is usually considered logistically simpler but it is still not well studied whether it gives the same results (Szefer et al. 2017, but see e.g. recent study of Guo et al. (2024)). The decomposability of dried fresh material should be higher than for litter but, since certain structural and chemical traits are preserved during the resorption process, they are expected to be positively correlated (Cornelissen et al. 1999; Guo et al. 2024).

## Methods

### Selection of species and sampling sites

We selected four *Carex* species (subgenus *Carex*) which are commonly found in meadows of the South Bohemian region of the Czech Republic: *Carex pallescens* L*.*, *C. panicea* L*.*, *C. pilulifera* L*.,* and *C. caryophyllea* Latourr. Each species was collected from four different meadows varying in environmental conditions. Due to shifted optima in ecological preferences between species (*C. caryophyllea* prefers drier habitats), only one site contained all four selected species (Table 1). Hence, a total of seven sites were sampled and contained one to four species (Table 1). We have already demonstrated that the species differ consistently in their leaf traits, even when grown under standardized conditions, but the intraspecific trait variability is large (Janíková et al. 2024).

**Table 1:** Description of sampling sites where *Carex* species were collected from. Table includes the name of the closest settlement, site coordinates and elevation, climatic data (mean annual temperature (MAT) and precipitation (MAP)), vegetation and management type, measured soil nutrient content, unweighted Ellenberg-type indicator values averaged for each site (M = moisture, T = temperature, N = site fertility, i.e. nutrients, L = light), and presence of *Carex* species at each location (*C. car* = *C. caryophyllea, C. pal* = *C. pallescens, C. pan* = *C. panicea, C. pil* = *C. pilulifera*). Climatic data was obtained from MeteoBlue website (www.meteoblue.com) and calculated between years 2010–2020. Vegetation type was determined with considering alliance identification in Probabilistic Vegetation Key (Tichý and Chytrý 2019) by the most abundant species recorded in relevés in field

Locality	Longitude	Latitude	Elevation (m)	MAT (°C)	MAP (mm)	Vegetation type	Management	Soil nutrient content						Ellenberg-type indicator values					*Carex* species			
								C (%)	N (%)	P (%)	K [mg kg ^−1^]	Ca [mg kg ^−1^]	Mg [mg kg ^−1^]	M	T	N	L	pH	*C. car*	*C. pal*	*C. pan*	*C. pil*
Bošice	49.088N	13.835E	623	8.9	806	Violion caninae, with elements of Molinion	mowing (1/year)	5.25	0.53	0.11	123	2520	542	5.5	4.9	5.0	7.0	5.0		x	x	
Ohrazení	48.953N	14.593E	517	9.5	715	Molinion caeruleae	mowing (1–2/year)	5.18	0.47	0.06	148	873	250	6.7	5.0	3.9	6.7	4.4		x	x	x
Tejmlov	49.132N	13.652E	900	9.5	715	Violion caninae	not managed	7.67	0.62	0.08	276	825	186	5.5	5.0	4.2	6.8	4.4			x	x
Úhřice	49.078N	13.921E	650	8.1	921	Violion caninae	mowing (1/year)	5.30	0.43	0.14	180	1011	211	4.5	5.5	4.1	7.0	5.2	x	x		x
Vrcov	48.921N	14.663E	495	9.5	698	Molinion caeruleae	mowing (1/year)	4.96	0.47	0.06	229	1266	230	5.3	5.1	4.9	6.9	5.4	x	x	x	x
Závraty	48.937N	14.378E	470	9.5	715	Arrhenatherion elatioris, with elements of Violion	mowing (1/year)	6.79	0.57	0.12	305	1236	229	4.2	5.6	4.3	7.2	5.6	x			
Zvíkov	48.989N	14.608E	510	9.5	715	Arrhenatherion elatioris	mowing (1/year)	6.17	0.54	0.10	101	2133	651	4.4	5.5	4.3	7.0	5.6	x			

The sampled meadows varied from mesic to wet, mowed at least once a year (material is collected in the mown plots) apart from Tejmlov, where no mowing occurred (Table 1). We used Ellenberg-type indicator values to describe differences in local environmental conditions between sampling sites. This approach relies on the ecological preferences of the species composing the plant communities (Chytrý et al. 2018). Based on three vegetation relevés (2 m × 2 m), we recorded the list of all vascular plants per sampling site, extracted 5 Ellenberg-type indicator values (moisture, nutrient, light, temperature, and pH) for all species from Chytrý et al. (2018), and calculated their unweighted mean values. A principal component analysis was used to summarize the variation in the 5 Ellenberg-type indicator values among sampling sites and demonstrated that sites were primarily differentiated by the first axis corresponding to moisture, light and pH, and the second independent axis to nutrients (Fig. S1). Because the first axis was mainly determined by moisture (also confirmed by our field experience), with clear differences in moisture among localities, we decided to use indicator values for moisture and nutrients. Indeed, the correlation between moisture and light was −0.91, and if used as explanatory variable, the results were nearly identical to those with moisture.

### Microcosm experiment

In late November 2020, at the peak of plant senescence, 20 individuals per species were harvested in each sampling site. Individuals were marked earlier in July to ensure correct species identification. Standing litter was separated from fresh leaves and samples of both leaf types were prepared for a decomposition assay. Individuals were combined to form 10 pairs of samples per population (litter and fresh leaves) of 50 to 100 mg of dry mass each. This number was reduced to four and eight pairs for *C. pallescens* collected in Úhřice and Ohrazení, respectively, due to the small size of individuals. *Carex* samples were dried at room temperature and stored in paper bags until the beginning of the experiment.

Laboratory microcosms were prepared following Kuebbing et al. (2018) and Güsewell and Gessner (2009). Each microcosm consisted of a 240 mL jar half filled with 40 g of sterilized sand (2 mm diameter) and covered with a piece of fine mesh to protect samples from contaminations. Dried *Carex* leaves were cut to *cca.* 5 cm long pieces and evenly placed on top of the mesh. A 17 mL solution of soil inoculum was added to each jar. This inoculum was prepared from a mixture of fresh soil collected in all sampling sites, diluted in distilled water using a 1:10 ratio, and filtered through a 50 µm filter.

In early December, a total number of 298 microcosms were randomly placed in a climabox at 15 °C and constant humidity (around 70%). Wet clothes on top of the jars were used to maintain high humidity in the microcosms and jars were watered occasionally with distilled water to compensate for evaporation. After 80 days, when we estimated *cca.* 30% mass loss (i.e. the initial phase of nutrient loss) on average in all samples, all jars were harvested, and the remaining biomass was dried at 60 °C for 48 h and weighed. We calculated the decomposition rate (*k* values) for each sample using the exponential decay equation (Aerts and de Caluwe 1997):

$${w}_{t}={w}_{0}{e}^{-kt}$$ or $$\text{ln}\left(\frac{{w}_{t}}{{w}_{0}}\right)=-kt$$, thus $$k=-\frac{\text{ln}\left(\frac{{w}_{t}}{{w}_{0}}\right)}{t}=-\frac{\text{ln}(1-\frac{WL}{100})}{t}$$

where w_0_ and w_t_ are the initial and final sample dry mass, respectively; *t* is time measured in years; and *k* is the decomposition rate [yr^−1^]. *WL* is a percentage weight loss, sometimes alternatively used as a measure of decomposition rate and shows that *k* is a simple log function of it (with eighty days exposition time, 30% weight loss corresponds to *k* = 1.63). Thus, the results based on weight loss are very similar (shown in Table S1).

### Trait measurements

Leaf area, specific leaf area (SLA) and leaf dry matter content (LDMC) were measured on living plants in May 2020 of the same populations, at the peak of growing season, following standard protocols (Pérez-Harguindeguy et al. 2013): 10 healthy individuals were selected per species and per study site, and two fully developed and undamaged leaves were selected on each plant individual. Leaves were scanned to measure leaf area, then fresh and dry mass were determined respectively. SLA corresponds to the area of leaf per unit dry mass [mm^2^ mg^−1^] and LDMC to the ratio between the dry and the water-saturated leaf fresh mass [mg g^−1^].

Chemical analyses were conducted on the samples harvested in November 2020. Material from several individuals was combined randomly to produce 3 pairs of samples per population (fresh leaves and litter) that were used for the chemical analyses. Analyses were conducted at the Analytical Laboratory of the Institute of Botany CAS. Total C and N [% in dry matter] were determined by dry combustion with Thermo Scientific FLASH 2000 Elemental Analyzer, and total P [% in dry matter] with QuikChem® 8500 flow analyzer (Lachat Instruments, Loveland, USA) after mineralizing with perchloric acid. Ca, Mg and K was extracted using Mehlich III extractant solution (Mehlich 1984), and concentrations [mg kg^−1^] were determined by atomic absorption spectrophotometry (AAS Spectrometer ContrAA 700, Analytik Jena, Jena, Germany). C:N and N:P ratios were calculated from obtained data. Due to the small amount of available leaf material, only C, N and P content could be determined in 13 samples, and one population (*C. pilulifera* from Vrcov) is missing from the analysis due to the same reason. Principal component analyses were conducted to show the variations and covariations in chemical properties in the whole dataset and in fresh leaves and litter separately (Figs. S2–S4).

### Data analysis

Statistical analyses were carried out in R version 4.0.3 (R Core Team 2021). ANOVAs were used to test the effect of the leaf type (fresh leaves and litter), species identity, sampling sites and their interactions on *k* values, leaf traits, and initial leaf nutrient content (data did not require transformation). The same analyses were conducted for each leaf type separately. Tukey- and Sidak-tests were performed to compare *k* values of species within leaf types and populations within species (‘multcomp’ and ‘emmeans’ packages; Hothorn et al. 2008; Lenth 2024).

We tested the consistency of the correlations between *k* values, Ellenberg-type indicator values, leaf traits (measured at fresh leaf level) and initial nutrient content, both across species and within species between populations. To do so, Pearson’s correlation coefficients and their significance were calculated within and between species using the ‘statsBy’ function (‘psych’ package, Revelle 2024). The same analysis was conducted to test the relationship between *k* values of dried fresh leaves and litter. In the latter, groupings were done both at the species and population levels. Since the results are based on a hierarchical structure and provide compound significance for among and within species, which is difficult to visualize, in the figures we present significance based on simple correlations between *k* and the tested variables for each line separately.

Figures were created with ‘ggplot2’ (Wickham 2016), ‘ggpubr’ (Kassambara 2020) and ‘factoextra’ (Kassambara and Mundt 2020).

## Results

### Leaf decomposition rates

**Fig. 1:**
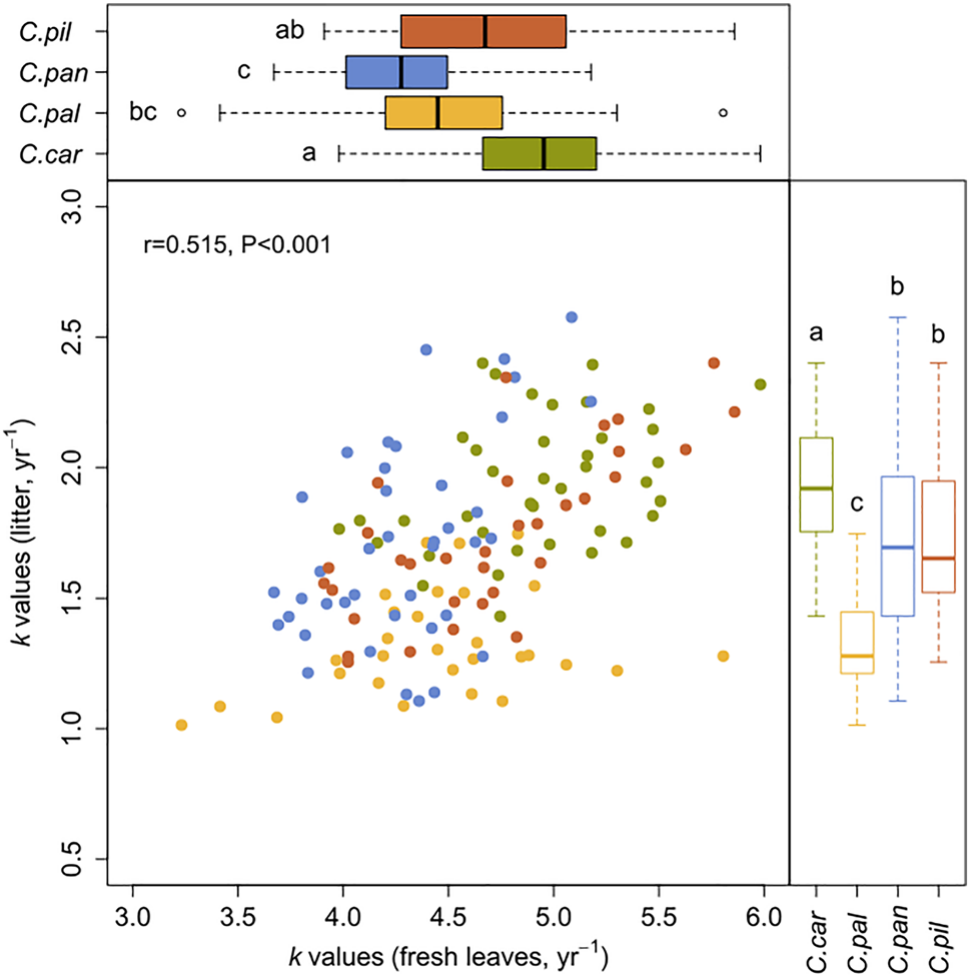
Relationship between decomposition rates (*k* values) of fresh leaves and leaf litter combined with distribution of *k* values for the 4 *Carex* species. In the middle panel each point represents a sample pair (green – *C. caryophyllea*, yellow – *C. pallescens*, blue – *C. panicea*, orange – *C. pilulifera*). On the sides each box displays the first quartile, median, third quartile, whiskers refer to 1.5 interquartile range and open circles are additional outliers in the data. Filled boxplots represent green leaf tissues, open boxplots with coloured outlines refer to leaf litter. Different letters indicate significant differences between species according to Tukey-test at *P* < 0.05

**Fig. 2:**
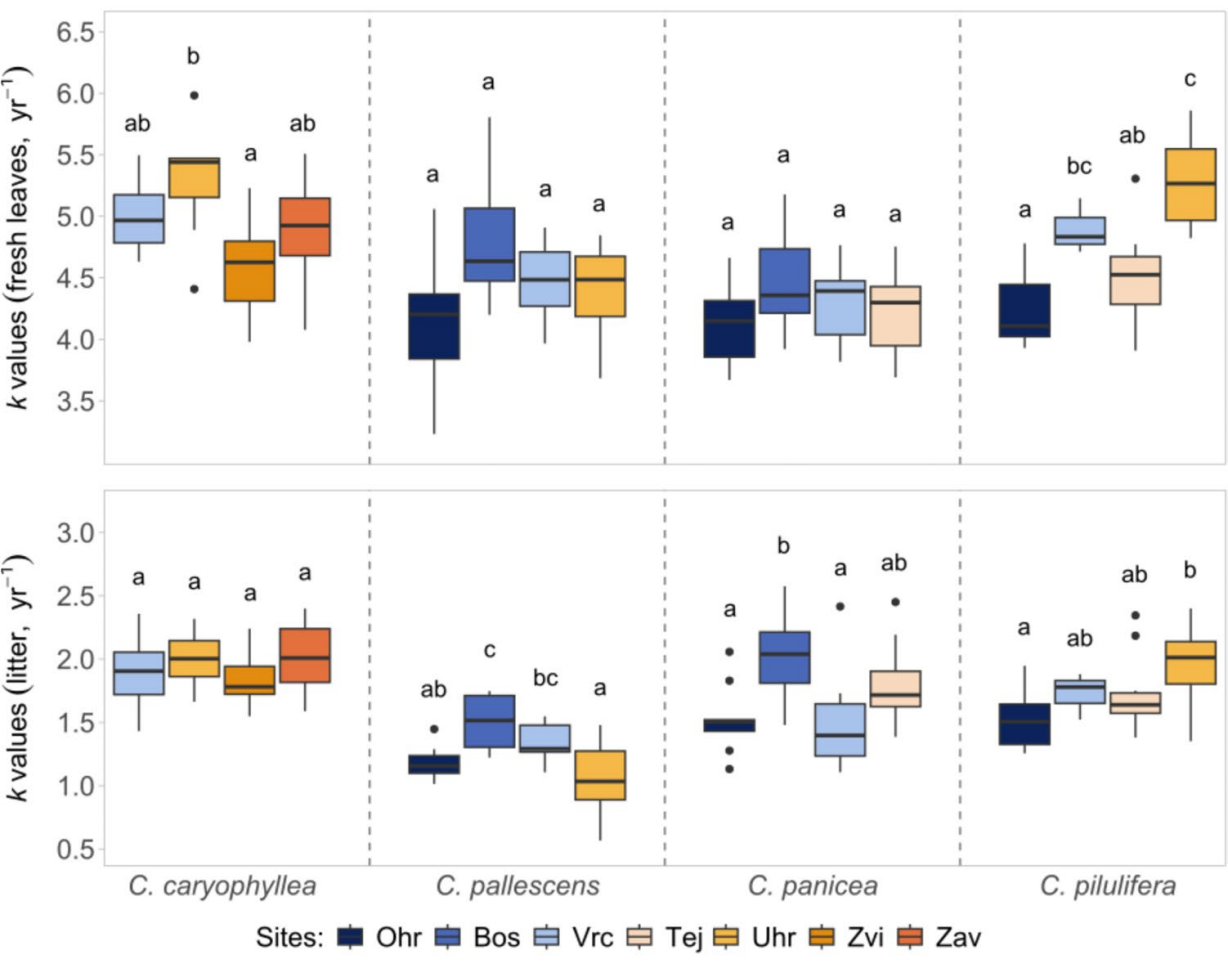
Differences in decomposition rate (*k* values) between populations of *Carex* species. Each box displays the first quartile, median, third quartile, whiskers refer to 1.5 interquartile range and black points are additional outliers in the data. In total there are 16 boxplots, corresponding to one of the 16 species × site combinations. Site abbreviations are the following: Ohr – Ohrazení, Bos – Bošice, Vrc – Vrcov, Tej – Tejmlov, Uhr – Úhřice, Zvi – Zvíkov, Zav – Závraty. Sites are ordered and colored from the wettest (Ohrazení) to the driest (Závraty) according to unweighted Ellenberg-type moisture indices calculated for sampling sites. Different letters indicate significant differences between sites within species according to Sidak-test at *P* < 0.05

The rate of decomposition differed between species in the following order: *C. caryophyllea* > *C. pilulifera* > *C. pallescens* > *C. panicea* in fresh leaf samples, and *C. caryophyllea* > *C. pilulifera* ~ *C. panicea* > *C. pallescens* in litter (Fig. 1 and Table S2). When considering all variables together, leaf type had the most pronounced effect on *k* values, followed by the species identity (considering *F-*values as a measure of effect size, Table 2). The interaction between these two factors was also significant, showing that the difference between litter types changes with the species: for example, the *k* value was 2.5 times higher in fresh leaves than in litter in *C. caryophyllea*, but this difference was increased to more than 3.5 times in *C. pallescens* (Table S2). The effect of the sampling site was also significant; however, it explained considerably less variation in *k* than species identity (Fig. 2, Table 2). The effect of populations (species × site interaction) was even weaker: it explained a tenth of the variability compared to species identity, nonetheless it was still significant. Within each leaf type, the effect of species and sites separately were also substantial, but the effect of populations was not consistent (Fig. 2, Table S3).

**Table 2:** Effect of leaf type (litter and fresh leaves), species identity, sampling site and their interactions on decomposition rate (*k* values [yr^−1^]) of dried *Carex* leaves, tested by ANOVA

	df	*Mean Sq*	*F*	*P*
Type	1	622.96	5509.37	** < 0.001**
Species	3	4.94	43.66	** < 0.001**
Site	6	1.42	12.52	** < 0.001**
Type × Species	3	1.24	10.96	** < 0.001**
Species × Site	6	0.46	4.10	** < 0.001**
Type × Site	6	0.45	4.02	** < 0.001**
Type × Species × Site	6	0.10	0.84	0.534
Residuals	258	0.11		

Bold numbers represent significant results (*P* < 0.05), *F* = F-values, df = degrees of freedom

We confirmed that fresh leaves decomposed faster than litter (average *k* = 4.60 yr^−1^ for fresh leaves and *k* = 1.63 yr^−1^ for litter) and that the two measures were positively correlated (r = 0.51, *P* < 0.001, Fig. 1); positive correlations were also confirmed both among and within species (Fig. 1, Table 3).

**Table 3:** Pearson’s correlation coefficients (r) and probabilities (*P*) between decomposition rates (*k* values) of fresh leaves and litter between and within groups. At the species level, grouping of variables was done by species (4 species replicated 4 times), while on population level grouping was executed by the number of populations (16 populations replicated 3 to 10 times)

	Between groups		Within groups	
	r	*P*	r	*P*
Species level	0.603	0.397	**0.687**	**0.005**
Population level	**0.632**	**0.009**	**0.370**	** < 0.001**

Bold values represent significant results

### Effect of environmental conditions and traits on decomposition

**Fig. 3:**
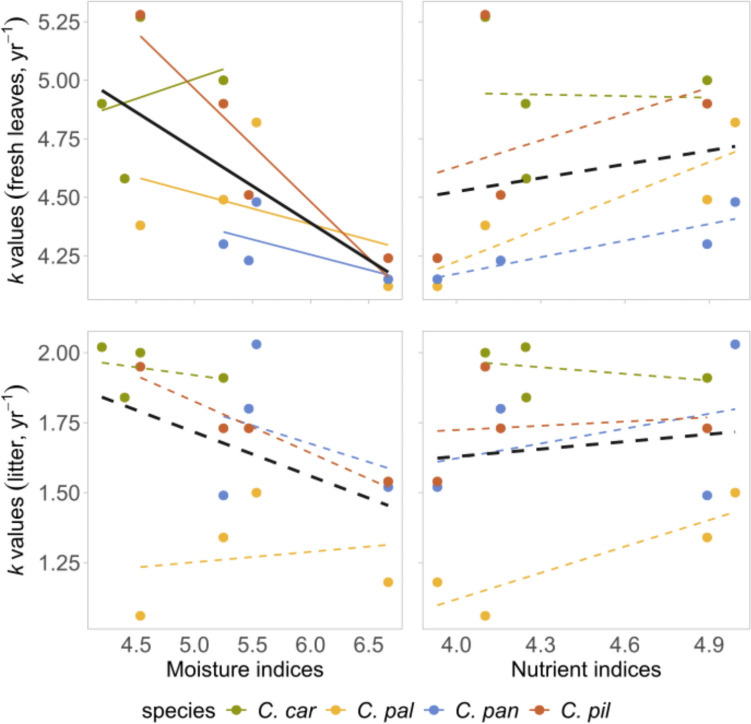
Correlation of Ellenberg-type moisture and nutrient indices on decomposition rates (*k* values) for fresh leaves and litter of different *Carex* species (*C. car* = *C. caryophyllea, C. pal* = *C. pallescens, C. pan* = *C. panicea, C. pil* = *C. pilulifera*). Each point is equivalent to a single sample. Correlations were calculated for all samples together (black line), and for different species (lines corresponding to species’ colors). Dotted lines represent non-significant, full lines represent significant relationships (*P* < 0.05). Note the different scales on the y axis for the fresh leaves and litter

**Fig. 4:**
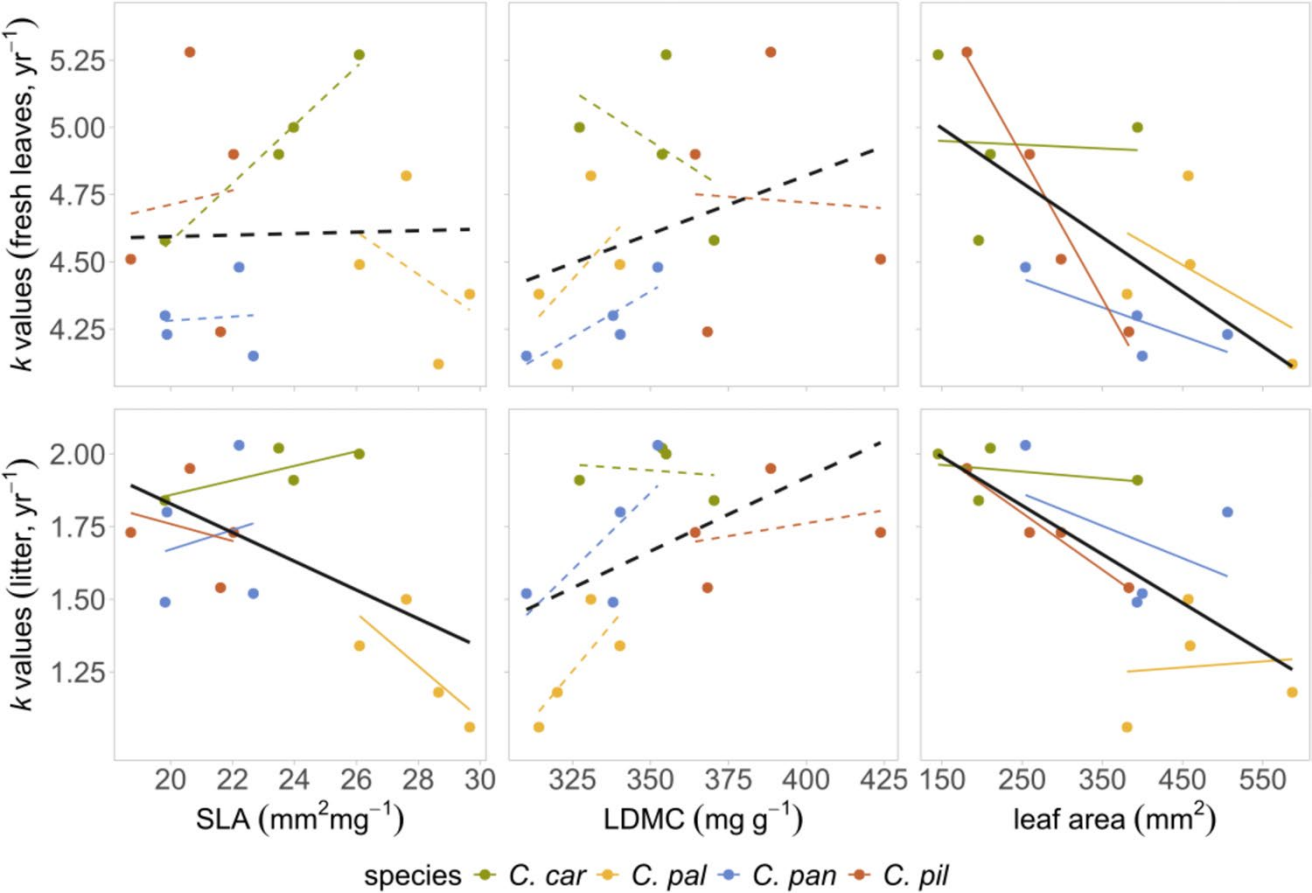
Correlation of mean decomposition rate (*k* values) and leaf traits (SLA, LDMC and leaf area) measured for fresh leaves and litter of different *Carex* species at the peak of growing season (explanation of species names in Fig. 3). Each point represents one of the 16 species × site combinations. Correlations were calculated for all samples together (black line), and for different species (lines corresponding to species’ colors). Dotted lines represent non-significant, full lines represent significant relationships (*P* < 0.05). Note the different scales on the y axis for the fresh leaves and litter

Overall, we found little evidence for relationships between site environmental conditions and *k* values of both leaf types and at both intra- and interspecific scales. We only found a significant negative effect of site moisture on fresh leaf *k* within species, and differences in site fertility had no effect on *k* values (Table 4 and Fig. 3). While all leaf traits measured in spring significantly differed between species and sampling sites (Fig. 4, Table S4), they generally had no significant effect on *k* (Table 4). Only a significant negative relationship was found with leaf area in fresh leaf decomposition within species (Table 4).

**Table 4:** Pearson’s correlation coefficients (r) and probabilities (*P*) of decomposition rate (*k* value) and Ellenberg-type indicator values, leaf traits and leaf nutrients between and within species. ‘Leaf type’ refers to correlations between different leaf types (‘fresh’ = fresh leaf decomposition rates correlated with corresponding fresh leaf characteristics, ‘litter’ = litter decomposition rates correlated with corresponding litter characteristics)

		Between groups		Within groups	
trait	leaf type	r	*P*	r	*P*
Moisture indices	fresh	−0.871	0.129	**−0.544**	**0.036**
	litter	−0.566	0.434	−0.330	0.220
Nutrient indices	fresh	−0.744	0.252	0.458	0.082
	litter	−0.495	0.505	0.407	0.127
SLA (mm^**2**^ mg^**−1**^)	fresh	−0.122	0.878	0.272	0.315
	litter	−0.753	0.247	−0.020	0.942
LDMC (mg g^**−1**^)	fresh	0.587	0.413	0.034	0.900
	litter	0.524	0.476	0.426	0.109
leaf area (mm^2^)	fresh	−0.842	0.158	**−0.525**	**0.044**
	litter	−0.918	0.082	−0.379	0.157
C:N ratio	fresh	0.048	0.952	−0.324	0.228
	litter	0.226	0.774	−0.103	0.718
N:P ratio	fresh	0.425	0.575	−0.158	0.562
	litter	0.343	0.657	0.005	0.985
K (mg kg^−1^)	fresh	0.004	0.996	−0.310	0.250
	litter	0.868	0.132	**0.617**	**0.022**
Ca (mg kg^−1^)	fresh	0.522	0.478	0.389	0.145
	litter	0.732	0.268	0.148	0.616
Mg (mg kg^−1^)	fresh	−0.319	0.681	0.335	0.213
	litter	0.280	0.720	0.353	0.220

Bold values represent significant correlations. Ellenberg-type indicator values (moisture and nutrient indices) and leaf traits (SLA, LDMC, leaf area) were only available for living individuals from field measurements for comparison with *k* values

**Fig. 5:**
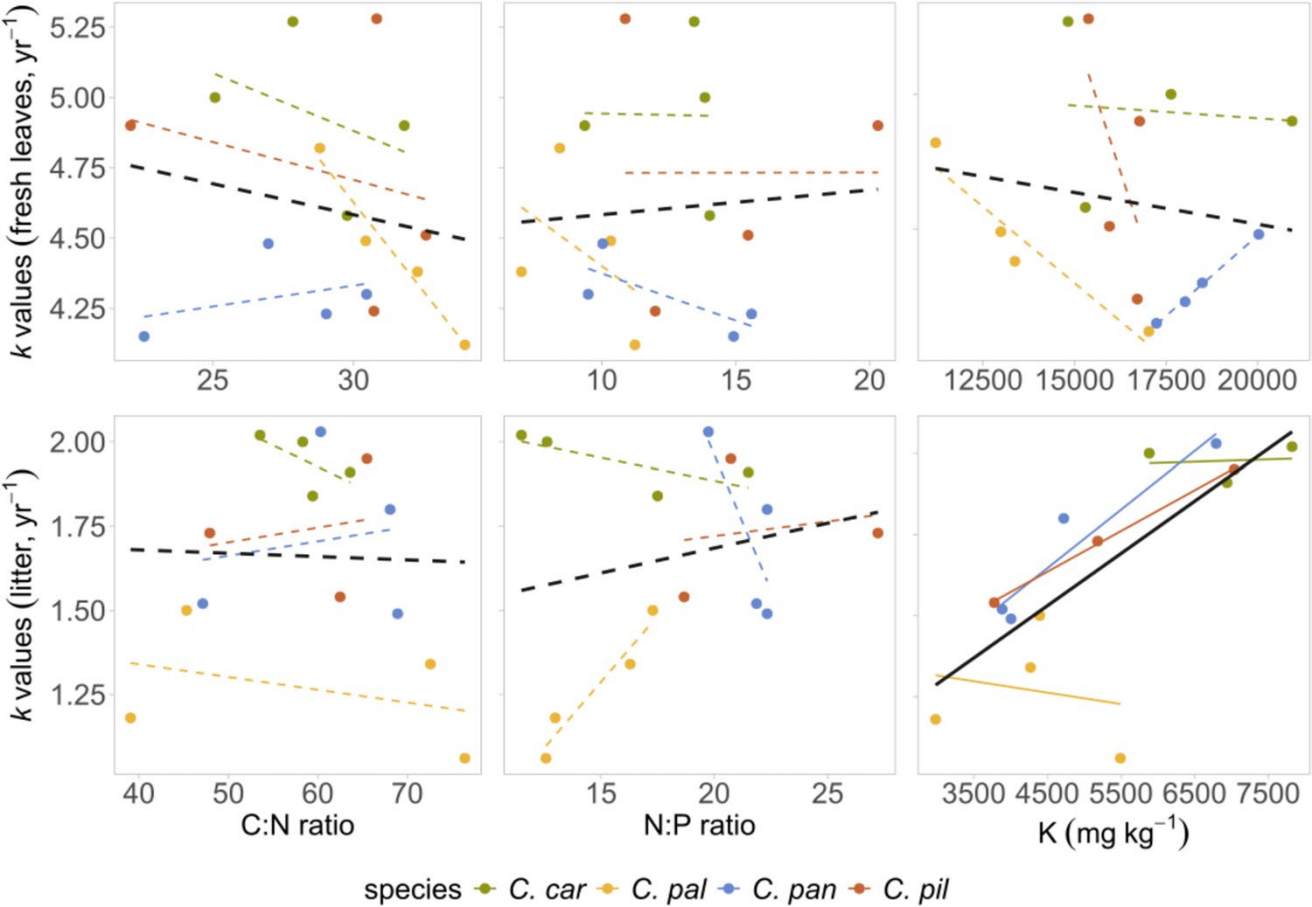
Correlation of mean decomposition rate (*k* value*)* and C:N, N:P ratios and K concentration in fresh leaves and litter of different *Carex* species (explanation of species names in Fig. 3). Selection of leaf nutrients was executed according to first axis scores of principal component analyses. Each point represents one of the 16 species × site combinations. Correlations were calculated for all samples together (black line), and for different species (lines corresponding to species’ colors). Dotted lines represent non-significant, full lines represent significant relationships (*P* < 0.05). Note the different scales on the y axis for the fresh leaves and litter, and the range of nutrient content on the x axis

As expected, the initial chemical composition of litter significantly differed from the fresh leaves (Table S5): for example, C:N ratio was on average twice as high in litter than in fresh leaves, K concentration was on average three times higher in fresh leaf samples compared to litter (Fig. 5). Species generally differed in chemical composition within fresh leaves as well (Table S6a), however in litter samples these differences mostly disappeared (Table S6b). Again, correlations of chemical composition with *k* values within leaf types were usually weak (Fig. 5), only K concentration had a significant effect in litter within species (Table 4).

## Discussion

### Between and within species variability of decomposition rates

Even though we executed the experiment on closely related species, we found that species identity had a predominant effect on leaf decomposability. Within the *Carex* genus, all belong to the same *Carex* subgenus, but within the *Carex* subgenus, they are phylogenetically relatively distant (based on node age values of a dated ultrametric supertree for Central European vascular plant species (DaPhnE 1.0, Durka and Michalski 2012)), suggesting that they might have had enough time in evolutionary context to differentiate their traits. This might also be the reason why these species are often found coexisting in nature under varying environmental conditions. Similarly to our study, Crutsinger et al. (2009) also compared different species and genotypes of *Solidago* in a decomposition experiment and found an overwhelming effect of species identity on litter decay, preceding genotype identity and genotypic diversity. In a broader scale decomposition experiment including long-term chronosequences, Wardle et al. (2009) also reported a prominent effect of species on litter mass loss during decomposition, compared to intraspecific differences. While we observed significant differences of decomposition rates among populations of the same species, and even though their effect was a fragment of species identity’s, it was nonetheless still significant (Table 2). Further, although we found consistency in decomposition rates of different populations within species, there were occasional shifts in the ranking between species (Fig. 2). For example, while litter samples of *C. pallescens* from Vrcov did decompose faster on average than samples from Úhřice (light blue and yellow boxes respectively), the order changes for *C. pilulifera*.

### Effects of environmental conditions and traits on decomposition

Our results generally showed very weak correspondence of decomposition rates with the environmental variables and leaf traits examined during the experiment. Though species samples were collected from various environmental conditions and their leaf traits measured at the peak of vegetation period also differed, there was little to no effect of these factors on leaf decomposability, contrary to our expectation based on the ‘afterlife’ effect hypothesis (Cornelissen et al. 2004; Cortez et al. 2007). Although *Carex* populations did differ initially in e.g. LDMC values (Table S4) or in C:N ratio (Table S5), these traits in the end did not contribute to explaining the decomposability of leaf material. Our results might thus imply that within these closely related species, the conditions of individual localities only modify negligibly, if at all, the tissue in a way that is changing their decomposability. This is also supported by the total effect of populations on the decomposition rate, which was a tenth of the effect size of species identity in general, but nevertheless still significant. Although the non-significant correlations of environmental variables and leaf traits with decomposability might also reflect low power of the test due to the low number of species and populations involved, the values of the correlation coefficients are rather low.

While environmental variables and leaf traits showed no or weak significant effects on leaf decomposition in our study, their overall importance should not be neglected. In the case of Ellenberg-type indicator values, moisture requirements seemed to have the strongest effect on decomposability in fresh leaves, and *k* values tended to decrease with the moisture index (Fig. 3). In leaf traits, leaf area proved to be the only trait showing a (rather weak) correlation with decomposition rates. We found a general negative trend in SLA and a positive trend with LDMC related to decomposability, which was also a rather unexpected finding. Previous studies report either a positive (e.g. Cornelissen et al. 1999; Sundqvist et al. 2011; Szefer et al. 2017; Liu et al. 2018; De La Riva et al. 2019) or no correlation (Cornelissen 1996, including *C. flacca* among selected monocots) between SLA and litter decomposability, and a negative correlation with LDMC and leaf decomposability (e.g. Fortunel et al. 2009; Sundqvist et al. 2011; De La Riva et al. 2019). However, most of the studies are executed on the community level, including a wide range of plant species and life forms, while our observations only cover a handful of closely related species. Another possible explanation of this phenomenon might be driven by the effect of *C. pallescens*, that gave the lowest *k* values despite having the highest SLA values and low LDMC (Fig. 4). On the other hand, we also have to consider that using fresh leaves to compare for litter *k* might not give a realistic idea of the actual correlation, due to the initial differences in material quality. Our results could also imply that some of the nutrients have already been leached in situ before sample collection happened in autumn, which might also explain the unexpected lack of correlations between species *k* values and most leaf traits.

Principal component analyses on leaf nutrients showed that the variability was mainly covered by C:N and N:P ratios and leaf cations (Figs. S2-S4). However, except for K concentration in litter none of the leaf nutrients showed a significant correlation with *k* values. Several studies concluded that higher base cation content can be positively correlated with faster decomposition of litter (see e.g. Cornelissen and Thompson 1997; Pálková and Lepš 2008). *Carex* species generally contain a relatively high amount of foliar K in fresh leaves (Busch 2001), but the amount of water-soluble Ca is relatively low (Hawkesford et al. 2012). It seems however that Ca functions as a cation promoting nutrient resorption in fresh leaves, while during senescence it is retained from other organs to the leaves (Jonasson and Chapin 1985; Cornelissen and Thompson 1997).

### Differences of decomposition rates between leaf types

As expected, fresh leaves were decomposing much faster than leaf litter did: during the 80 days of the experiment ~ 30% of the litter material decayed, as compared to ~ 50% of fresh leaves. The correlation between the decomposition rates of the two leaf types proved to be rather strong, and was observed both among species, among populations within species and among individuals. The non-significant correlation between species is simply because with four species, the power of the test is extremely low, but still r ~ 0.6. This is in alignment with the recent findings of Guo et al. (2024), who also observed a strong correlation in *k* between fresh leaves and leaf litter across different life forms (including a wider range of herbs: r^2^ = 0.60, *P* < 0.01) in their study. Fresh leaves generally possess different characteristics than litter, which can alter the speed and overall quality of decomposition (Guo et al. 2024). The principal component analyses also pointed towards differences in chemical composition between fresh leaves and litter (Figs. S2-S4). It is also likely that the litter collected in situ is less “standardized” (i.e. in different stages of senescence) than the fresh leaves, which might play a role in comparison of similar closely related species*.* This might be especially pronounced in *Carex* species and other sedges that usually develop new leaves while retaining nutrients from older ones, gradually producing standing dead litter (Jonasson and Chapin 1985; Shaver and Laundre 1997). Leaves decomposing faster also lose their decomposable substances already on the plants (Sanaullah et al. 2010), and thus the collected litter decomposes slower. Differences in resorption capacity (i.e. the amount of decomposable substances already lost) before the litter is collected might also vary among species and among individuals of the same population, introducing another level of uncontrolled variation. Species might retrieve their nutrients in different ways, and this might be the reason why there are significant differences among species in both leaf types, however not in an identical order. One of the reasons of the different nutrient retrieval might also be in the large differences in the tendency to form rhizomes, which we have demonstrated for our experimental species (Janíková et al. 2024). In conclusion, even though fresh leaves might be a good proxy to predict litter decomposition rates, using litter material is a better way to predict real decomposability in nature, when feasible.

## Conclusions

We demonstrated that intraspecific differences in decomposition rates of four *Carex* species, both within and between sites, were significant. However, interspecific differences in decomposition rates were more pronounced than differences among individual populations. The variation in decomposability between sites did not follow any clear trend, neither with the Ellenberg-type indicator values of localities, nor with the individual populations. According to our expectations, fresh leaves decomposed much faster than the litter collected in situ, and their values were well correlated. Nevertheless, the pattern of inter- and intraspecific variation in *k* values was rather different in fresh leaves and litter.

## Supplementary Information

Below is the link to the electronic supplementary material.

Supplementary file1 (DOCX 247 kb)

## Data Availability

The datasets used and/or analysed during the current study are available from the corresponding author on reasonable request.
