# Supplementary material for: Intraspecific differences in leaf decomposition and associated traits in closely related Carex species: a microcosm experiment
Source: Oecologia. 2025 Jun 12;207(7):102. doi: 10.1007/s00442-025-05740-1 (PMC12162743; doi:10.1007/s00442-025-05740-1)
Supplement: Supplementary file 1 — Supplementary file1 (DOCX 247 kb) [file 442_2025_5740_MOESM1_ESM.docx]

Electronic Supplemental Material for

**Intraspecific differences in leaf decomposition and associated traits in closely related *Carex* species: a microcosm experiment**

Szilvia Márta Neumann^1*^, Jules Segrestin^1^, Marie Konečná^1^, Aleš Lisner^1^, Markéta Applová^1,2^, Petr Blažek^1^, Anna E-Vojtkó^2^, Eva Janíková^1^, Lars Götzenberger^1,2^ & Jan Lepš^1,3^

*^1^Department of Botany, Faculty of Science, University of South Bohemia, České Budějovice, Czech Republic*

*^2^Institute of Botany, Czech Academy of Sciences, Třeboň, Czech Republic*

*^3^Biology Center of the Czech Academy of Sciences, Institute of Entomology, České Budějovice, Czech Republic*

***Corresponding author**, e-mail: [szilvia.neumann@gmail.com](mailto:szilvia.neumann@gmail.com)

**Table S1.** Effect of leaf type (litter and fresh leaves), species identity, sampling site and their interactions on weight loss [*%*] of dried *Carex* leaves, tested by ANOVA. Bold numbers represent significant results (*P* < 0.05), *F* = F-values, df = degrees of freedom.

|  | df | *Mean Sq* | *F* | *P* |
| --- | --- | --- | --- | --- |
| Type | 1 | 77514 | 5448.34 | **<0.001** |
| Species | 3 | 633 | 44.47 | **<0.001** |
| Site | 6 | 164 | 11.56 | **<0.001** |
| Type × Species | 3 | 179 | 12.60 | **<0.001** |
| Species × Site | 6 | 38 | 2.65 | **0.017** |
| Type × Site | 6 | 25 | 1.76 | 0.108 |
| Type × Species × Site | 6 | 10 | 0.72 | 0.634 |
| Residuals | 258 | 14 |  |  |

**Table S2.** Mean values (±SD) of decomposition rates (*k* values [yr^-1^]) calculated for the four *Carex* species, decomposed to fresh leaves and litter. Bold numbers represent the highest, bold italic numbers the lowest mean values in each column.

| **species** | ***k* values (fresh leaves)** | ***k* values (litter)** |
| --- | --- | --- |
| *C. caryophyllea* | **4.93±0.44** | **1.94±0.25** |
| *C. pallescens* | 4.45±0.53 | ***1.25±0.26*** |
| *C. panicea* | ***4.29±0.37*** | 1.71±0.39 |
| *C. pilulifera* | 4.70±0.55 | 1.74±0.31 |

**Table S3.** Effect of species identity, sampling site and their interaction on decomposition rates (*k* values [yr^-1^]) in dried fresh leaves and litter respectively, tested by ANOVA. Bold numbers represent significant results (*P* < 0.05), *F* = F-values, df = degrees of freedom.

|  | **fresh leaves** | | | **litter** | | |
| --- | --- | --- | --- | --- | --- | --- |
|  | df | *F* | *P* | df | *F* | *P* |
| Species | 3 | 19.36 | **<0.001** | 3 | 42.77 | **<0.001** |
| Site | 6 | 9.49 | **<0.001** | 6 | 5.69 | **<0.001** |
| Species × Site | 6 | 2.10 | 0.058 | 6 | 3.06 | **0.008** |
| Residuals | 125 |  |  | 133 |  |  |

**Table S4.** Effect of species, site and their interaction on leaf traits (SLA [mm^2^ mg^-1^], LDMC [mg g^-1^], leaf area [mm^2^]), measured at the peak of vegetation growth on the same populations that were sampled for decomposition, tested by ANOVA. Bold numbers show significant results (*P* < 0.05), *F* = F-values, df = degrees of freedom.

|  |  | **SLA (mm^2^ mg^-1^)** | | **LDMC (mg g^-1^)** | | **leaf area (mm^2^)** | |
| --- | --- | --- | --- | --- | --- | --- | --- |
|  | df | *F* | *P* | *F* | *P* | *F* | *P* |
| Species | 3 | 27.45 | **< 0.001** | 31.28 | **< 0.001** | 25.05 | **< 0.001** |
| Site | 6 | 3.00 | **0.009** | 6.24 | **< 0.001** | 7.06 | **< 0.001** |
| Species × Site | 7 | 1.33 | 0.239 | 3.68 | **0.001** | 1.69 | 0.116 |
| Residuals | 151 |  |  |  |  |  |  |

**Table S5.** Effect of leaf type, species, sampling site and their interactions on C:N and N:P ratios, and Ca, Mg and K concentration [mg kg^-1^] in dried *Carex* leaves, tested by ANOVA. Bold numbers show significant results (*P* < 0.05), *F* = F-values, df = degrees of freedom.

|  |  | **C:N ratio** | | **N:P ratio** | |  | **K (mg kg^-1^)** | | **Ca (mg kg^-1^)** | | **Mg (mg kg^-1^)** | |
| --- | --- | --- | --- | --- | --- | --- | --- | --- | --- | --- | --- | --- |
|  | df | *F* | *P* | *F* | *P* | df | *F* | *P* | *F* | *P* | *F* | *P* |
| Type | 1 | 365.02 | **< 0.001** | 78.53 | **< 0.001** | 1 | 596.81 | **< 0.001** | 159.33 | **< 0.001** | 11.21 | **0.002** |
| Species | 3 | 0.13 | 0.943 | 12.85 | **< 0.001** | 3 | 7.78 | **< 0.001** | 2.23 | 0.098 | 1.17 | 0.332 |
| Site | 6 | 5.10 | **< 0.001** | 4.40 | **0.001** | 6 | 2.16 | 0.066 | 2.75 | **0.024** | 4.85 | **< 0.001** |
| Type × Species | 3 | 0.94 | 0.426 | 3.75 | **0.016** | 3 | 5.28 | **0.004** | 1.22 | 0.312 | 4.28 | **0.010** |
| Type × Site | 6 | 5.07 | **< 0.001** | 1.07 | 0.393 | 5 | 1.41 | 0.241 | 0.26 | 0.930 | 2.45 | **0.049** |
| Species × Site | 6 | 2.47 | **0.035** | 2.24 | 0.053 | 6 | 1.43 | 0.225 | 0.75 | 0.616 | 0.95 | 0.472 |
| Type × Species × Site | 5 | 3.40 | **0.010** | 0.73 | 0.603 | 5 | 0.90 | 0.488 | 0.78 | 0.573 | 2.46 | **0.048** |
| Residuals | 55 |  |  |  |  | 43 |  |  |  |  |  |  |

**Table S6**. Effect of species identity, sampling site, and their interaction on C:N and N:P ratios, and Ca, Mg and K concentration [mg kg^-1^] of dried fresh leaves (Table S6a) and litter (Table S6b), tested by ANOVA. Bold numbers show significant results (*P* < 0.05), *F* = F-values, df = degrees of freedom.

| **a)** |  | **C:N ratio** | | **N:P ratio** | |  | **K (mg kg^-1^)** | | **Ca (mg kg^-1^)** | | **Mg (mg kg^-1^)** | |
| --- | --- | --- | --- | --- | --- | --- | --- | --- | --- | --- | --- | --- |
|  | df | *F* | *P* | *F* | *P* | df | *F* | *P* | *F* | *P* | *F* | *P* |
| Species | 3 | 4.63 | **0.009** | 12.68 | **< 0.001** | 3 | 15.77 | **< 0.001** | 13.16 | **< 0.001** | 5.19 | **0.007** |
| Site | 6 | 2.30 | 0.061 | 7.74 | **< 0.001** | 6 | 3.25 | **0.019** | 4.77 | **0.003** | 1.70 | 0.169 |
| Species × Site | 6 | 3.70 | **0.007** | 5.40 | **< 0.001** | 6 | 2.44 | 0.059 | 1.41 | 0.256 | 2.88 | **0.032** |
| Residuals | 30 |  |  |  |  | 22 |  |  |  |  |  |  |

| **b)** |  | **C:N ratio** | | **N:P ratio** | |  | **K (mg kg^-1^)** | | **Ca (mg kg^-1^)** | | **Mg (mg kg^-1^)** | |
| --- | --- | --- | --- | --- | --- | --- | --- | --- | --- | --- | --- | --- |
|  | df | *F* | *P* | *F* | *P* | df | *F* | *P* | *F* | *P* | *F* | *P* |
| Species | 3 | 0.15 | 0.93 | 6.84 | **0.002** | 3 | 1.58 | 0.224 | 0.52 | 0.675 | 0.55 | 0.652 |
| Site | 6 | 4.89 | **0.002** | 1.82 | 0.136 | 5 | 1.00 | 0.441 | 1.25 | 0.323 | 5.85 | **0.002** |
| Species × Site | 6 | 2.81 | **0.038** | 0.84 | 0.536 | 5 | 0.45 | 0.808 | 0.72 | 0.614 | 0.61 | 0.692 |
| Residuals | 30 |  |  |  |  | 21 |  |  |  |  |  |  |


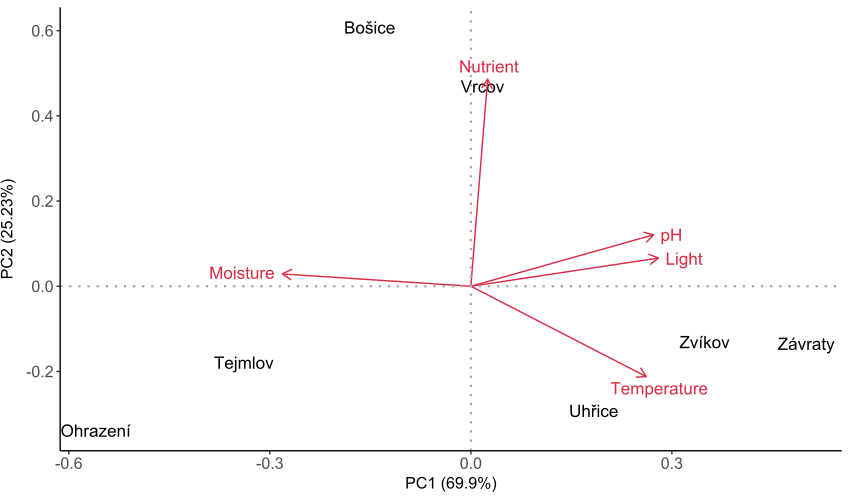


**Fig. S1.** Principal component analysis on Ellenberg-type indicator values (EIV) calculated from relevé data from each sampling site (moisture, nutrient, light, temperature and pH indices). The first axis is distinctly defined by moisture requirements of species together with temperature, pH and light requirements, while the second axis is explained by nutrients.


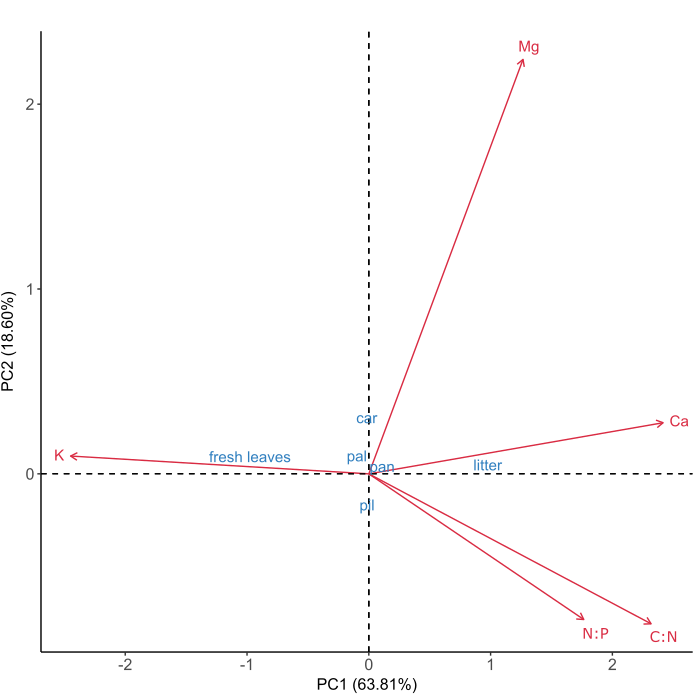


**Fig. S2.** Principal component analysis on leaf nutrients with leaf type and species (‘car’= *C. caryophyllea*, ‘pal’= *C. pallescens*, ‘pan’= *C. panicea,* ‘pil’ = *C. pilulifera*) as supplementary variables. The first axis clearly defines a difference in chemical composition between leaf types, i.e. higher C:N and N:P ratio paired with low K concentration in litter, and the opposite in fresh leaves, while species align more along the second axis.


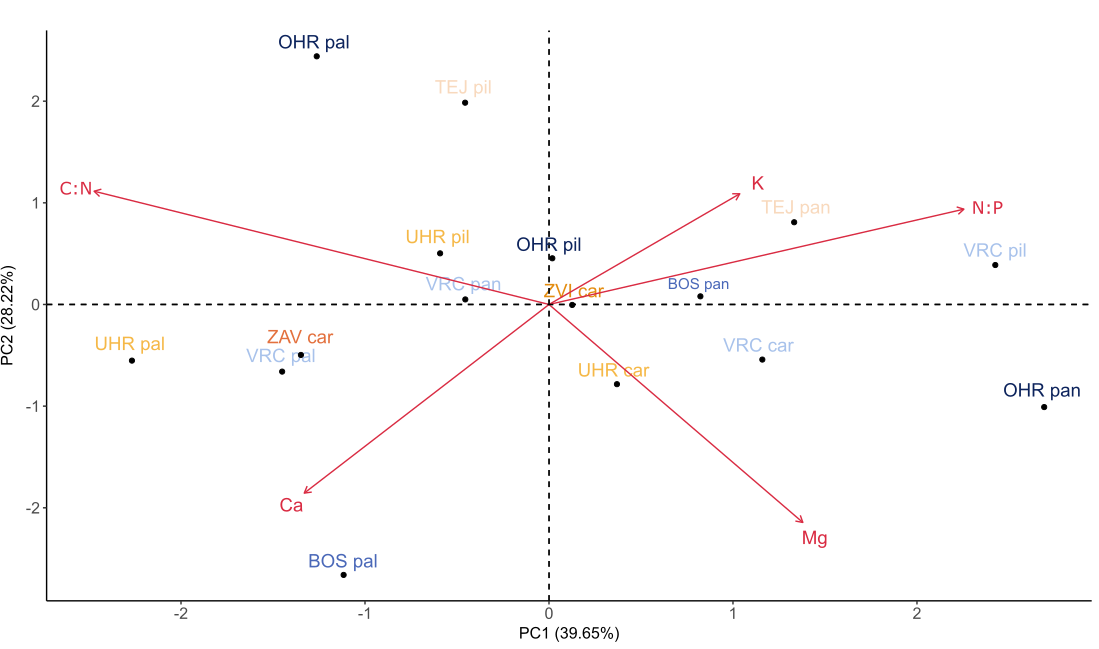


**Fig. S3.** Principal component analysis on leaf nutrients in fresh leaf samples, including all populations (16 points) in the ordination space. Populations are colored according to unweighted Ellenberg-type moisture indices calculated for each site, analogous with Figure 2. In population names uppercase abbreviations refer to sampling sites, while lowercase abbreviations refer to species names.


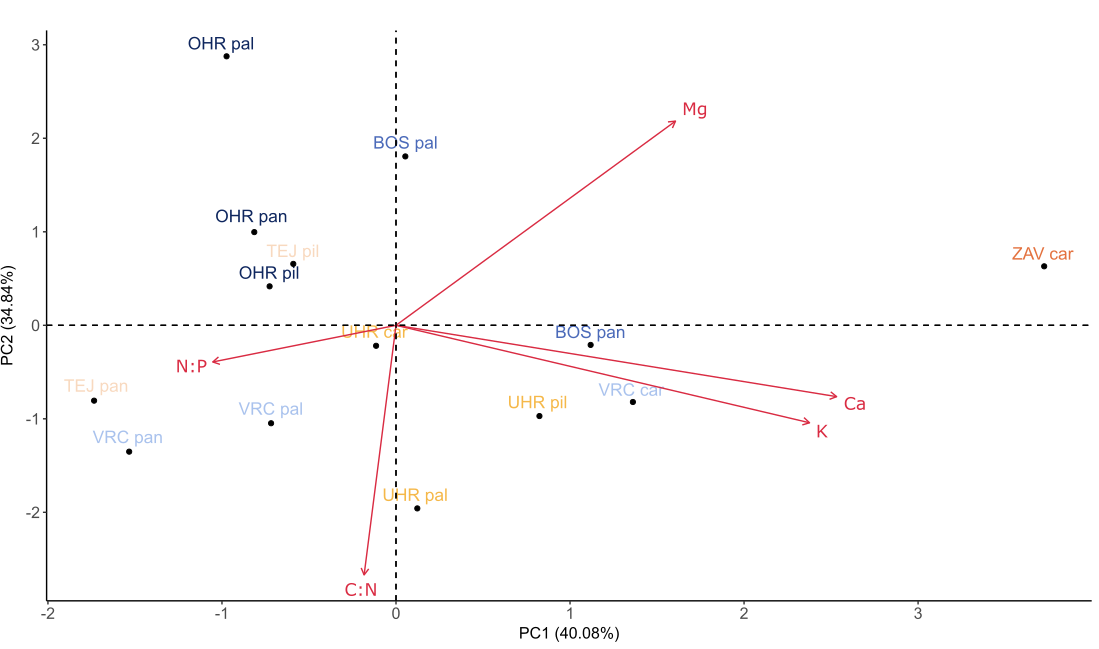


**Fig. S4.** Principal component analysis on leaf nutrients in litter samples, including all examined populations in the ordination space. Populations are colored according to unweighted Ellenberg-type moisture indices calculated for each site, analogous with Figure 2. In population names uppercase abbreviations refer to sampling sites, while lowercase abbreviations refer to species names. Two populations (VRC pil and ZVI car) were missing from the analysis due to the availability of plant material (14 points in total). Notable, that the relation of N:P and C:N ratios, and N:P ratio and Ca to Mg and K changes in litter compared to fresh leaves in Fig. S3.
